# Lidocaine infusion for the treatment of complex regional pain syndrome: Case series and literature review

**DOI:** 10.1515/med-2024-1102

**Published:** 2024-12-03

**Authors:** Kevin J. Yang, Porus D. Mistry

**Affiliations:** Keck School of Medicine, University of Southern California (USC), Los Angeles, United States of America; Department of Anesthesiology, Keck Medicine of the University of Southern California (USC), Los Angeles, United States of America

**Keywords:** causalgia, chronic pain, complex regional pain syndrome, lidocaine, neuropathic pain, reflex sympathetic dystrophy

## Abstract

**Introduction:**

Complex regional pain syndrome (CRPS) is a chronic pain condition most often triggered by direct injury to an extremity that is characterized by disproportionate pain, sensory abnormalities, and autonomic dysfunction. Early research into intravenous lidocaine therapy for CRPS has demonstrated promise, but clinical evidence remains scarce. We report on 12 patients with chronic CRPS who underwent intravenous lidocaine therapy and discuss our findings in the context of the existing literature.

**Results:**

Patients ages ranged from 25 to 64 years. Duration of CRPS ranged from 4 to 25 years. The majority of patients (8/12, 67%) reported adequate subjective pain relief with intravenous lidocaine therapy, whereas four patients reported inadequate subjective pain relief with therapy. All patients were being treated with at least one other pharmacotherapy. Three patients experienced minor side effects.

**Conclusions:**

Our cases, taken with existing evidence, suggest that intravenous lidocaine for the treatment of chronic CRPS is safe and may decrease the pain associated with chronic CRPS. However, this study lacks adequate sample size to make those conclusions confidently. We recommend a randomized placebo-controlled multicenter clinical trial to establish the efficacy and side effect profile of systemic intravenous lidocaine more confidently for the treatment of pain due to chronic CRPS.

## 1 Introduction

Complex regional pain syndrome (CRPS) is a chronic pain condition characterized by disproportionate pain, sensory abnormalities, and autonomic dysfunction. CRPS is most often triggered by direct injury to an extremity, including trauma and surgery. The pathophysiology of CRPS is thought to result from the interplay of neuroinflammatory, vascular, and central nervous system (CNS) mechanisms. Dysregulation of the sympathetic nervous system, neurogenic inflammation, and maladaptive plasticity in the CNS contribute to the perpetuation of pain and sensory symptoms observed in CRPS [1]. Many CRPS patients present with the acute form, which often self-resolves within a year of inciting injury. However, some patients will progress to the chronic form, in which patients suffer from long-term debilitating pain [1].

CRPS is classified into two subtypes: Type I, formerly known as reflex sympathetic dystrophy and Type II, previously termed causalgia. Type I CRPS occurs without identifiable nerve injury, whereas Type II CRPS is associated with confirmed nerve damage [1]. Both types manifest with similar clinical features, including severe pain, edema, changes in skin temperature, and motor dysfunction. To aid in diagnosis of CRPS, the Budapest criteria, established by the International Association for the Study of Pain, provides a standardized framework encompassing clinical signs and symptoms characteristic of CRPS [2].

While various treatment modalities exist for chronic CRPS management, ranging from physical therapy to pharmacotherapy and interventional procedures, a significant proportion of patients experience inadequate pain relief or intolerable side effects [3]. For refractory cases, therapeutic options are limited, emphasizing the urgent need for novel therapies.

Preclinical studies have demonstrated promising results regarding the efficacy of intravenous lidocaine infusion in alleviating CRPS-related pain and symptoms. Those studies uncovered that, subsequent to injury of peripheral nerves, there is an augmentation in tetrodotoxin-resistant sodium channels within primary nociceptive afferent fibers alongside minute dorsal root ganglia neurons involved in pain transmission, resulting in notable physiological alterations [4–6]. The infusion of lidocaine intravenously obstructs these particular channels, hindering repetitive depolarization in a manner contingent upon usage [7].

Those findings have prompted further exploration of lidocaine as a potential therapeutic agent in human subjects. Prior randomized placebo-controlled trial of intravenous lidocaine to treat neuropathic pain (5 mg/kg/h over 6 h) demonstrated 4 h pain relief after cessation of the infusion without causing adverse side effects [8]. A prior retrospective review of 49 patients who underwent intravenous lidocaine (titrated to a lidocaine blood concentration of 5 mg/L of blood delivered daily over the course of 5 days) for treatment of chronic, refractory CRPS demonstrated a significant decrease in mechanical and thermal allodynia for 3 months, lessened associated inflammatory components of CRPS, minimal side effects, and no severe complications [9].

Clinical evidence supporting the use of lidocaine infusion in CRPS management remains scarce. As such, we aim to add to the body of evidence evaluating the effectiveness of lidocaine infusion as a therapy for the treatment of CRPS. We present a case series of patients who underwent systemic intravenous lidocaine infusion for the treatment of chronic CRPS and report on whether each patient experienced subjective pain relief. We then report on our literature review of intravenous lidocaine for the treatment of chronic CRPS and discuss our findings in the context of the existing literature.

## 2 Methods

### 2.1 Chart review

This study was a retrospective chart review. A list of all past and present patients who were treated with intravenous lidocaine at the University of Southern California (USC) Pain Center’s associated intravenous infusion center was obtained. The medical indication for each of these patient’s intravenous lidocaine therapy were found by reviewing each patient’s electronic medical records. Those patients whose indication for intravenous lidocaine therapy was CRPS were included in the study.

For those patients included in the study, whether or not the patient’s follow-up visit records included a qualitative description of positive response to lidocaine infusion was recorded; this parameter was used as our measure of subjective adequate pain relief. Other data collected are presented in Section 3. Inclusion was limited to patients who met the Budapest criteria for CRPS. This study was reviewed and determined to be exempt §46.104(d) (4) on 4 March 2024 by the USC Institutional Review Board. This study was performed in accordance with CARE guidelines. All patient details have been de-identified.

### 2.2 Patient treatment

All patients first underwent electrocardiogram, complete metabolic panel, and magnesium level testing prior to initiation of lidocaine infusion therapy. During their first infusion session, all patients were started on a lidocaine infusion dose of 2 mg/kg/h, administered over 4 h. If tolerated, the dosage was increased to a goal of 4 mg/kg/h the following day. All subsequent infusion sessions were 4 h in duration. Subsequent infusions were repeated on a schedule of two consecutive days of infusion per month at the tolerated dose. During all infusion sessions, patients were continuously monitored by infusion unit nurses with a physician immediately available at all times. All patients were provided standard of care treatment, had full information regarding expected benefits and possible complications of intravenous lidocaine therapy. All patients gave informed consent to treatment.

### 2.3 Literature review

A comprehensive literature review was conducted to identify relevant studies on the use of intravenous lidocaine for the treatment of CRPS. The search was performed exclusively using the PubMed database (https://pubmed.ncbi.nlm.nih.gov/). The search strategy aimed to capture all studies evaluating IV lidocaine in the context of CRPS management. The following search query was used to retrieve relevant articles: (“lidocaine”[Title/Abstract] OR “intravenous lidocaine”[Title/Abstract]) AND (“complex regional pain syndrome”[Title/Abstract] OR “CRPS”[Title/Abstract]). This query was designed to include both key terms for lidocaine and CRPS, accounting for potential variations in terminology. No filters were applied to limit publication date or study type to ensure a broad inclusion of available literature. Articles identified through the search were screened based on their relevance to the topic, and studies specifically investigating the use of intravenous lidocaine for treating CRPS were selected for inclusion in the review.

## 3 Results

We found a total of 12 patients being treated for CRPS by intravenous lidocaine infusion. Patient ages ranged from late 20s to 60s. Nine of our patients were female and three were male. Duration of CRPS ranged from 4 to 25 years. Eight patients reported adequate subjective pain relief with intravenous lidocaine therapy, whereas four patients reported inadequate subjective pain relief with therapy. Three patients experienced minor side effects. One patient experienced perioral numbness at infusion rate of 4 mg/kg/h, which resolved on reduction of the infusion rate to 3 mg/kg/h. One patient experienced mental fogginess when their infusion rate was increased from 2 to 4 mg/kg/h, which resolved on reduction of the infusion rate to 3 mg/kg/h. One patient experienced dizziness during one infusion session, which self-resolved over the course of the infusion. Patient demographics, details about patient CRPS condition, details about patient lidocaine infusion therapy parameters, response to therapy, and complications associated with therapy are reported in Table 1. Our data revealed no clear relationship between number of infusions, lidocaine dosage, CRPS duration, and adequate pain relief.

**Table 1:** Patient demographics, details about CRPS condition, details about lidocaine infusion, response to lidocaine infusion, and complications of therapy

Patient number	Age	Sex	Site of pain	Duration of CRPS (years)	Number of infusions	Initial lidocaine dose (mg/kg/h)	Most recent lidocaine dose (mg/kg/h)	Adequate subjective pain relief?	If inadequate pain relief, change made	Complications
1	Late 40s	F	RUE, BLE	25	16	2	4	No	Added IV ketamine infusions	
2	Late 30s	F	RUE	5	5	2	4	Yes		
3	Late 30s	F	RUE, LUE	11	42	2	4	Yes		
4	Late 50s	F	RLE	20	10	2	4	No	Stopped lidocaine infusion	
5	Early 50s	F	RUE, BLE	14	19	2	4	Yes		
6	Early 40s	F	LLE	Unknown	5	2	4	Yes		
7	Late 20s	F	RUE	4	3	2	3	Yes		
8	Early 60s	F	Bilateral feet	7	25	2	3	Yes		Patient experienced perioral numbness at infusion rate of 4 mg/kg/h. Resolved on reduction of infusion rate to 3 mg/kg/h
9	Early 60s	F	Left knee	9	3	2	3	Yes		Patient experienced mental fogginess when uptitrated from 2 to 4 mg/kg/h. Subsequently reduced to 3 mg/kg/h
10	Late 50s	M	LLE	16	6	2	4	Yes		
11	Late 20s	M	LLE	7	4	2	4	No	Stopped lidocaine infusion	Dizziness (resolved during infusion)
12	Early 50s	M	BLE	9	2	2	4.75	No	Stopped lidocaine infusion	

Note: F = female, M = male, RUE = right upper extremity, RLE = right lower extremity, BLE = bilateral lower extremities, LUE = left upper extremity, LLE = left lower extremity, BUE = bilateral upper extremities, IV = intravenous, mg/kg/h = milligrams of lidocaine per patient’s weight in kilograms per hour of infusion.

All patients were being treated with at least one other pharmacotherapy for the management of CRPS-related pain at the time of initiating lidocaine infusion therapy. The additional pharmacotherapy of each patient is reported in Table 2.

**Table 2:** Additional pharmacotherapy for pain of each patient at time of lidocaine infusion therapy

Patient number	NSAID	Antidepressants	Anticonvulsants	Spasmolytics	Opioids	Oral ketamine	Transdermal ketamine	Intramuscular ketamine	IV Ketamine	Benzodiazepines	Transdermal lidocaine (patch)
1	Yes		Yes	Yes	Yes	Yes					
2		Yes	Yes	Yes	Yes	Yes			Yes		
3			Yes	Yes	Yes	Yes					
4	Yes	Yes		Yes	Yes	Yes				Yes	Yes
5		Yes		Yes		Yes	Yes		Yes		
6	Yes		Yes			Yes					
7								Yes			
8	Yes	Yes	Yes		Yes	Yes			Yes	Yes	Yes
9			Yes		Yes	Yes			Yes		
10		Yes			Yes						
11		Yes									
12		Yes			Yes						

Note: NSAID = non-steroidal anti-inflammatory drug, IV = intravenous.

## 4 Discussion

We report on 12 patients who underwent systemic intravenous lidocaine infusion for the treatment of chronic CRPS. Of our patients, the majority of them (8/12, or 67%) experienced clinically significant reduction in pain symptoms. While our sample size is not large enough to demonstrate a meaningful correlation between intravenous lidocaine therapy and pain relief, our pain relief findings are consistent with the two other currently published studies investigating the efficacy of systemic intravenous lidocaine for the treatment of CRPS.

Schwartzman et al. investigated the efficacy of systemic intravenous lidocaine titrated to lidocaine blood concentration of 5 mg/L of blood delivered daily over the course of 5 days for the treatment of chronic CRPS with a sample size of 49 patients [9]. Schwartzman et al. found improvement in dynamic and static mechano-allodynia, deep muscle pain, joint pain, and thermal allodynia (cold stimulus) in all patients at 3 months following infusion, with return to baseline by 6 months following infusion. Their study reports that the most profound improvement was on thermal and mechanical allodynia. They also reported a statistically significant but a much less robust improvement of movement symptoms of CRPS, including weakness, spasms, dystonia, and tremor.

Wallace et al. also investigated the efficacy of systemic intravenous lidocaine for the treatment of pain associated with CRPS Types I and II [10]. In their randomized, double-blind, placebo-controlled, crossover study, 16 patients received a systemic infusion of lidocaine. Spontaneous and evoked pain scores and neurosensory testing within the painful area were measured when patient plasma levels of lidocaine reached 1, 2, and 3 μm/mL of lidocaine and were compared to baseline measurements. All patients also underwent the same testing while undergoing intravenous diphenhydramine infusion (separated from lidocaine therapy by a washout period of 1 week) as a control. The authors found that lidocaine infusion caused a significant elevation of the hot pain thresholds in the affected area. Furthermore, the authors found a significant decrease in hyperalgesia when patients receiving lidocaine were stroked with a cotton wisp or exposed to cold. While this study reports statistically significant pain improvement in multiple forms, it fails to evaluate response to therapy for any significant amount of time following treatment, limiting the clinical significance of the authors’ findings.

The only other study uncovered by our literature search reporting on pain relief from systemic intravenous lidocaine in CRPS patients was a case report by Rickard and Kish [11]. These authors report on a patient with CRPS who underwent three intravenous lidocaine infusions in an intensive care unit setting, each providing significant pain relief. During the first admission, the patient received 1 mg/kg/h over 4 h. During the second admission, the patient underwent infusion at a rate of 1.5 mg/kg/h for 4 h, followed by a third infusion 3 days later at 2 mg/kg/h for 4 h.

Three patients in this study experienced minor adverse effects. One patient experienced perioral numbness, which resolved on reduction of the infusion rate from 4 to 3 mg/kg/h. One patient experienced mental fogginess when their infusion rate was increased from 2 to 4 mg/kg/h, which resolved on reduction of the infusion rate to 3 mg/kg/h. One patient experienced self-resolving dizziness during one infusion session. As such, the side effects reported by patients in this study were mild and temporary in nature. Of the side effects noted by Wallace et al. in their study, only light headedness was significantly higher in the lidocaine treatment group compared to the control group. It is possible that the “mental fogginess” and “dizziness” described by two of our patients is the same phenomenon as the “light-headedness” experienced by the Wallace et al. patients. Schwartzman et al. reported complications in 16 patients, all of which did not persist beyond 12 h after cessation of treatment. Most were mild and included, nausea, fatigue, mild cardiac side effects, mild neurological side effects, and mild psychiatric side effects. One patient experienced seizure. Of note, none of the complications reported by our patients were reported by Schwartzman et al. That fact, alongside the variety of complications reported by Schwartzman et al., suggest that complications of intravenous lidocaine therapy have widely variable presentations. All currently reported complications of lidocaine infusion therapy were benign.

All the patients included in this study were being treated with other medications at the time of initiating systemic intravenous lidocaine therapy. Concurrent treatment with various agents can theoretically complicate the use of systemic IV lidocaine in treating CRPS. Seven patients were being treated with antidepressants; tricyclic antidepressants in particular are known to increase the risk of cardiac arrythmias, which may potentially compound the cardiac side effects of intravenous lidocaine. Five patients were treated with spasmolytics, including tizanidine, which has hypotensive effects that could be exacerbated by lidocaine’s vasodilatory properties. The potential for those effects emphasizes the importance of cardiac monitoring during lidocaine infusion sessions. Spasmolytics, in addition to anticonvulsants, opioids, and benzodiazepine medications are known to have sedative and CNS depressive effects, which could be compounded by the sedative effect of intravenous lidocaine infusion. Those potential interactions suggest that CNS monitoring in such patients may be appropriate. It is important to note that such interactions are theoretical and merit further study.

Other forms of lidocaine administration for the treatment of CRPS-related pain have also been investigated. Several studies have examined using intravenous regional anesthesia (IVRA, also referred to as Bier block) techniques to administer lidocaine in solution with other agents for the treatment of CRPS in a specific extremity. Varitimidis et al. studied 168 patients with CRPS Type I of the upper extremity using a Bier block with 25 mL of 0.5% lidocaine and 125 mg of methylprednisolone, reporting significant improvements in pain relief, grip strength, and total active motion after an average of 4.8 sessions [12]. In a comparative study by Nascimento et al., IVRA using lidocaine and clonidine were shown to be as effective as sympathetic ganglion blocks for pain management in CRPS, though IVRA had fewer side effects and was easier to perform [13]. Eckmann et al. investigated the use of ketorolac and lidocaine for IVRA in patients with lower extremity CRPS, finding only short-term pain reduction, with pain typically returning within a week [14].

Topical lidocaine for CRPS pain management has also been explored. In a randomized placebo-controlled trial by Fallico et al., 150 patients were divided into three groups: lidocaine injections with oral citalopram, lidocaine injections with oral placebo, and placebo injections with oral placebo [15]. The combined treatment of lidocaine and citalopram significantly improved impairment sum scores (ISS) from 47.6 to 12.6, compared to lidocaine alone (ISS 47.5–21.5) and placebo (ISS 47.2–29.9), showing greater efficacy of the combined treatment over both placebo treatments. Additionally, a case report by Hanlan et al. described a patient with CRPS of the right upper extremity who experienced significant pain relief from topical lidocaine 5%, which allowed her to engage in physical therapy and improved her hand’s swelling and appearance over the course of her rehabilitation [16].

The reason why systemic intravenous lidocaine may be effective as therapy for CRPS may lie in lidocaine’s potential to modulate pain signaling pathways and suppress abnormal sensory processing. Lidocaine’s well-established efficacy as a local anesthetic results from the drug’s sodium channel-blocking activity, which transmit pain signals along the nerves. By inhibiting sodium channels, lidocaine dampens the transmission of pain signals from the affected area to the brain. Lidocaine has also been shown to block *N-*methyl-d-aspartate receptors [17], which contribute to the development and maintenance of chronic pain. By antagonizing these receptors, lidocaine may help reduce the hypersensitivity and central sensitization associated with CRPS.

Chronic inflammation has also been implicated in the pathophysiology of CRPS. The concentrations of proinflammatory cytokines, such as interleukin-1 (IL-1), IL-6, IL-8, and tumor necrosis factor (TNF)-α are elevated in the serum, cerebrospinal fluid, and skin blister fluid of CRPS patients [18]. Conversely, levels of anti-inflammatory cytokines such as IL-4, IL-10, and transforming growth factor-β are reduced in the serum of those patients [18]. Lidocaine has been shown to possess anti-inflammatory properties [19]. Specifically, lidocaine has been associated with a significant reduction in the following pro-inflammatory markers: TNF-α, IL-1RA, IL-8, IL-17, high mobility group box-1, and c-reactive protein [20]. As such, lidocaine may act by inhibiting the release of certain pro-inflammatory mediators associated with CRPS from immune cells and reducing the recruitment of inflammatory cells to the site of injury, thus reducing CRPS-related symptoms.

Furthermore, intravenous lidocaine has been reported to modulate sympathetic activity, possibly by blocking sympathetic ganglia or by modulating neurotransmitter release [21]. CRPS is characterized by dysregulation of the sympathetic nervous system, leading to abnormal vasoconstriction and other autonomic disturbances [22]. Thus, by blocking sympathetic ganglia or by modulating neurotransmitter release, intravenous lidocaine may alleviate the symptoms associated with sympathetic dysfunction in CRPS.

Lidocaine has also been shown to exert neuromodulatory effects on various neuronal circuits involved in pain processing [23]. By altering neuronal excitability and synaptic transmission, lidocaine may help normalize aberrant neural activity associated with chronic pain conditions like CRPS.

This case series, alongside the accompanying literature review, suggests that systemic intravenous lidocaine for the treatment of chronic CRPS is safe and may decrease the pain associated with chronic CRPS. However, this study lacks adequate sample size to make those conclusions confidently. High potential for bias and confounding factors in our dataset further limit the generalizability of this study. Given the present paucity of data in the existing literature, we recommend a randomized placebo-controlled multicenter clinical trial to establish the efficacy, duration of relief, and side effect profile of intravenous lidocaine more confidently for the treatment of pain due to chronic CRPS. Studies comparing the efficacy of systemic intravenous lidocaine to lidocaine administered by IVRA and topical lidocaine for the management of CRPS-related pain are also merited.
